# Study on Fatigue Life of Aluminum Alloy 6061-T6 Based on Random Defect Characteristics

**DOI:** 10.3390/ma17051133

**Published:** 2024-02-29

**Authors:** Lei Lu, Hao Chen, Mingming Ren, Sha Xu, Yongfang Li, Tianjun Zhou, Yali Yang

**Affiliations:** School of Mechanical and Automotive Engineering, Shanghai University of Engineering Science, Shanghai 201620, China; 18793506792@163.com (L.L.); 17856075700@163.com (M.R.); sxu@sues.edu.cn (S.X.); liyongfang@sues.edu.cn (Y.L.); zhoutianjun@sues.edu.cn (T.Z.); carolyn71@163.com (Y.Y.)

**Keywords:** CT scanning, random hole, 6061-T6 aluminum alloy, finite element simulation, fatigue life prediction

## Abstract

A certain number of hole-like defects will occur in aluminum alloys under cyclic loading. The internal holes will reduce the strength of the material and cause stress concentration, which will aggravate the development of fatigue damage. A classification method of defect features based on X-ray CT damage data is proposed. The random hole distribution model is established through the linear congruence method and the region division method. The hole parameter is introduced as the intermediate variable of the 3D reconstruction model of internal defects. In the mesoscopic stage, the function relationship between the distribution of random holes and the fatigue life is established based on the coupling relationship between the number and proportion of pores and the fatigue life. In the macroscopic stage, the relationship between the random holes and the macroscopic crack growth life is established by taking the crack length as the damage variable. The crack propagation rate decreased with the increase in the number of holes. The prediction model of the whole life stage is established using the life function from microcrack initiation to macroscopic crack propagation. Finally, the validity of the whole stage fatigue life prediction model is demonstrated through the comparison and verification of experiments, which provides a certain engineering value for the life estimation of 6061-T6 aluminum alloy materials.

## 1. Introduction

Aluminum alloys are widely used in the automotive industry, aerospace and other fields, due to their excellent corrosion resistance, easy processing and high strength characteristics [1,2]. However, aluminum alloys are prone to fatigue damage with the loading of cyclic stress. Studies have shown that more than 80% of fracture failures are caused by fatigue damage, so the fatigue damage of materials has always been the focus of scholars [3,4].

The fatigue failure of materials includes two stages: micro-crack initiation and macro-crack growth. In the crack initiation stage, there are many factors leading to micro-cracks, the most important of which is the hole defects formed during fatigue loading. The most important factor is the hole defects formed during fatigue loading. Wang et al. [5,6,7,8] demonstrated the effect of the characteristic parameters of hole defects on the generation and growth of material cracks and proposed a quantitative prediction model of fatigue life based on the interaction between holes. Deng et al. [9,10,11] studied the irregular distribution of hole state stress in material defects, revealed the failure law of randomly distributed holes on materials, and established a stress model for predicting fatigue life. Haridas et al. [12] used the probabilistic model as a prediction tool to estimate the fatigue life of the preferred microstructure with a lower pore number density and hole size. Chen et al. [13] established a prediction model between defects and fatigue life using the stress concentration factor as the intermediate variable of internal defects. Nourian et al. [14] established a crack growth model for fatigue life analysis using the average stress and characteristic parameters of defects. Wang et al. [15,16] established a fatigue life prediction model of aluminum alloys based on a neural network with the characteristic parameters of holes as the input. The aim was to analyze the characteristic parameters of the hole and the influence of the stress intensity factor on the fatigue life. However, the large number and uneven distribution of holes were not taken into account. In this paper, the classification of holes is simplified, and the establishment of a random hole model is based on the random distribution characteristics of holes. The functional relationship between the random hole parameters and the fatigue life at the micro-crack stage is obtained based on finite element simulation.

In the stage of macro-crack growth, many scholars have studied the mechanical parameters of crack growth. Chambel et al. [17] studied the fatigue crack growth under plane strain and plane stress loading and measured the fatigue crack growth rate (FCG) under cyclic loading. Gao et al. [18,19] established a fish-eye induced fracture model to predict fatigue life by using the initiation, growth and coalescence of cracks through high- and low-cycle fatigue cyclic loading tests. Correia et al. [20,21,22] studied the change in the Paris formula under different hole characteristics in the crack growth stage of materials and carried out finite element analysis and life prediction for crack growth with holes. Doh et al. [23] derived the fatigue life model using the Zhurkov life model, and verified it with the actual fatigue life data as a reference. The life prediction model above focuses on stress analysis at the crack. However, it is not related to the defect characteristics in the microcrack stage. This method lacks systematicness and has a single life prediction method. In this paper, the mapping relationship between the number of holes, crack length and number of cyclic loadings is established, and the life prediction function of macroscopic crack growth is constructed.

In summary, internal damage defects, fatigue crack initiation and propagation are regarded as damage evolution processes from the perspective of material damage evolution. The whole life prediction model of materials is constructed with damage defects as the intermediate variable. In addition, it also provides a new idea for fatigue life prediction of aluminum alloys.

## 2. Experimental Method

In this paper, the following parameters of the material were determined by means of the static tensile test: tensile strength, yield limit, elastic modulus E and so on. The basic parameters of the fatigue test were set using the mechanical parameters of static tension. The evolution law of fatigue damage was analyzed via CT scanning, and the defect characteristic information inside the material was obtained in order to construct a three-dimensional model with random holes as the damage characterization quantity. The prediction of fatigue life of materials with random holes was combined with finite element analysis, including microcrack initiation to crack growth failure under sub-regional conditions.

### 2.1. Materials and Specimens

**Figure 1:**
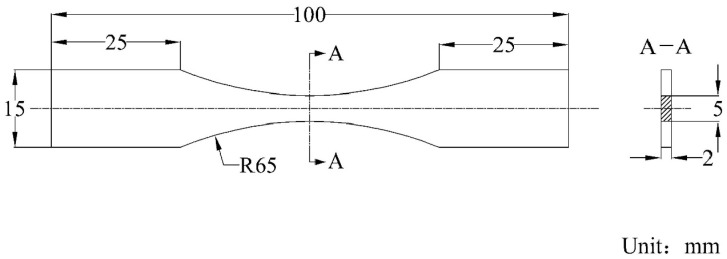
Specimen size diagram.

The material of the specimen selected in this paper was 6061-T6 aluminum alloy [24,25], and the main components are shown in Table 1. The preparation of the specimen was in accordance with the ASTM E1158-2014 standard [26]. The specimen size is shown in Figure 1.

### 2.2. Fatigue Test

Before the fatigue test, it was necessary to carry out a static tensile test on the material via an electronic universal tensile machine. A total of three test specimens were tested and averaged to obtain mechanical parameters [27], as shown in Table 2. The microcomputer-controlled electronic universal testing machine produced by Labsans company in Shenzhen, China, was adopted. The maximum tensile force was 100 KN, the effective tensile stroke of the test as 700 mm, and the constant tensile speed V = 0.2 mm/min.

**Figure 2:**
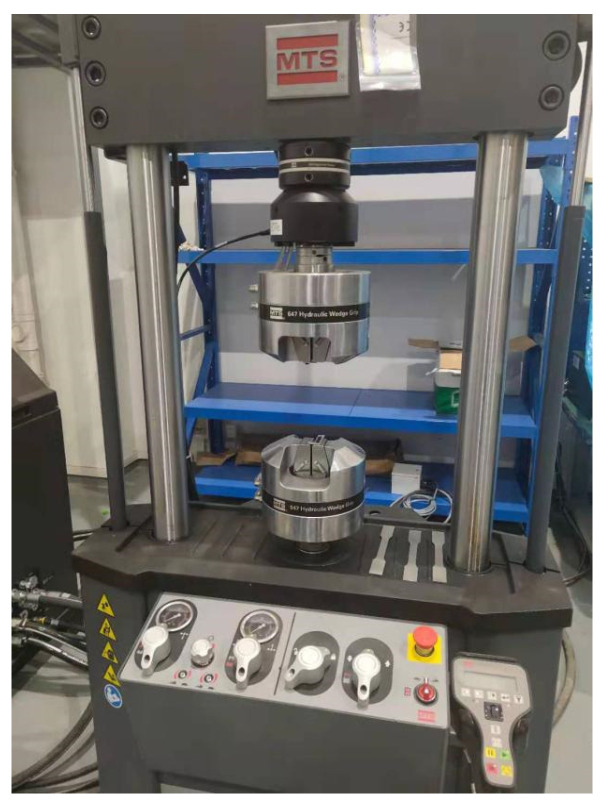
MTS fatigue testing machine.

**Figure 3:**
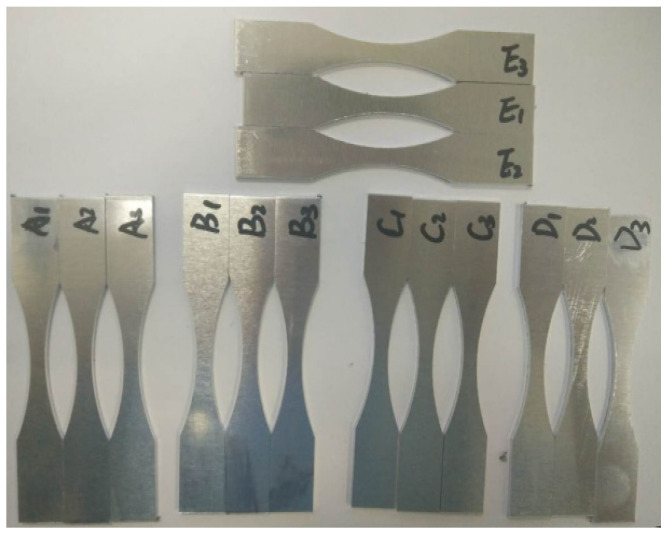
Fatigue tensile specimen.

The MTS hydraulic servo fatigue testing machine was used for fatigue cyclic test. The device is from MTS Corporation in Eden Prairie, MN, USA, as shown in Figure 2. The test was carried out at room temperature and the loading frequency was set to 50 Hz. The specimen was fixed at the left end and the cyclic load was applied at the right end. The stress ratio R was 0.1. The minimum load was 320 N and the maximum load was 3200 N. In order to obtain the damage conditions of the aluminum alloy in different loading stages, a total of 15 samples were tested for 40,000 cycles, 80,000 cycles, 120,000 cycles, 160,000 cycles and 200,000 cycles in cyclic loading tests, and 3 groups of repeated tests were performed at each loading stage, as shown in Figure 3. 

### 2.3. Data Processing of Defect Features

**Figure 4:**
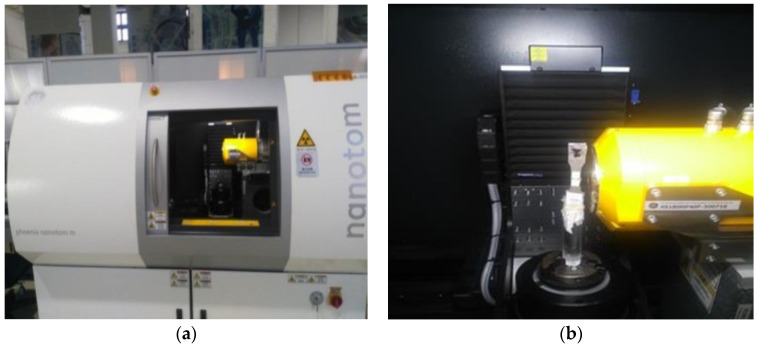
CT scanning device. (**a**) CT-Phoenix nanotom. (**b**) X-ray emitter.

An industrial CT-Phoenix nanotom manufactured by Phoenix, Germany, was used to study the evolution law of internal damage defects of specimens at different fatigue loading stages. A total of 15 fatigue specimens were X-CT scanned to obtain the gray level of two-dimensional tomography of the internal damage defects of the specimens [28]. The CT scanning equipment is shown in Figure 4.

The scanning voltage of CT equipment was set to 110 V, the current to 100 mA, the probe resolution to 2 μm, the exposure time to 1000 ms, and the number of two-dimensional fault gray maps was set to 2000 according to the size of the specimen. A computer system was used to reconstruct the 3D CT images from 2000 2D gray scale tomography images. The complete Tiff slice data of the scanning part of the specimen were obtained through VG STUDIO MAX 3.2 software. The hole information features were extracted from Tiff images using 3D visualization software AVIZO 9.2.0. The extracted hole defect parameters included hole radius, number, location and other information.

The experiment was divided into five cyclic loading stages. However, the material broke when stretched fewer than 200,000 times. Therefore, only the data for the four stretching stages from 4 to 16 million needed to be processed here.

**Figure 5:**
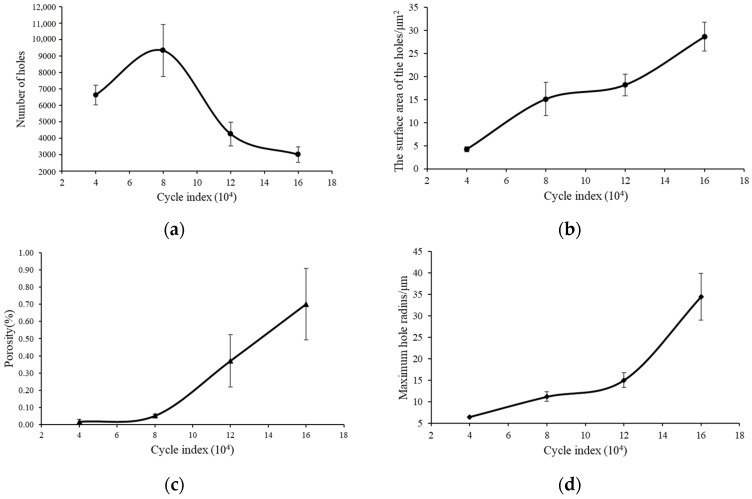
Relation between the characteristic parameters of holes and number of cycles. (**a**) Number of holes, (**b**) surface area, (**c**) porosity, (**d**) radius of holes.

Figure 5 is the change trend of the characteristic parameters of the hole with the number of cyclic loadings. It shows the standard error at the 85% confidence level. The trend of increasing pores in the material is more obvious when stretching is performed more than 80,000, as shown in Figure 5a. Internal holes begin to produce fusion when the specimen is stretched more than a certain number of times. At the same time, the fatigue damage of the material begins to occur, and the internal cracks begin to form and accelerate the growth. The continuous rise of the curve also indicates the continuity of fatigue damage.

Due to the discreteness of the test, there will be fluctuations in individual data. The overall surface area of the internal holes increases with the increase in the number of cycles, as shown in Figure 5b. The surface area of the hole can also be used as a damage variable to predict the remaining life of the material.

The inflection point of the porosity of the hole occurs when the number of cyclic loadings is 80,000. The porosity shows an upward trend, as shown in Figure 5c. Cracks begin to appear inside the material with continuous cyclic stretching. The hole radius increases dramatically when the number of cycles reaches 120,000. At this point, some of the small holes began to fuse into large holes, as shown in Figure 5d.

In summary, the influence of the holes on the fatigue life during the cyclic loading process has a strong relationship with their own characteristic parameters. These properties include hole radius, porosity, surface area, quantity, etc. However, it is necessary to build a simulation model in order to quantify the effect of the holes in the material on the fatigue life.

## 3. The Random Hole Model

The fatigue damage material contains a large number of approximate micro-hole defects with continuous cyclic loading, and the change in damage holes shows a certain regularity. The fracture process can be roughly divided into two stages. In the first stage, the holes inside the material gradually increase, and the volume gradually becomes larger until a failure crack is generated. This failure stage is the micro-crack initiation stage of the material. The second stage is the fatigue crack growth stage. The material begins to accelerate the growth, and experiences a large number of fatigue life cycles from crack initiation to complete fracture failure. These defect holes are randomly distributed inside the material in the actual situation. In order to more truly characterize the influence of hole characteristic parameters on fatigue life, it is necessary to establish a random hole model for research. 

### 3.1. Generation of Random Holes 

The generation of random numbers includes true random numbers and pseudo random numbers. A sufficient number of random numbers are generated based on the linear congruence method in pseudo-random numbers in MATLAB [29]. The recursive formula of this method is shown in Formula (1).

(1) $$X_{n+1}=(aX_{n}+c)\mathrm{mod}m$$

where X is a pseudo-random sequence. m(m>0), a(0<a<m), c(0≤c<m) are the modules, multipliers, and increments in the sequence, respectively. $X_{0}$ is the starting value of the sequence when n is 0.

Therefore, there are specific settings for parameters in the MATLAB platform library. where m is 2^32^, a is 8121, and c is 28,411. The pseudo-random number generator is often randomly tested in a sufficiently large interval, including parametric, independence and uniformity tests, and the three-dimensional coordinates of the holes can be randomly generated using this method.

### 3.2. Model Region Division

**Figure 6:**
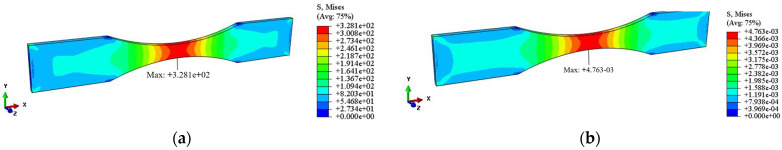
Stress analysis diagram (**a**) stress cloud diagram, (**b**) strain cloud diagram.

The software ABAQUS is used to analyze the stress of the complete specimen without holes. The mechanical parameters of the material are obtained via static tensile analysis. The stress and strain contours of the model without holes are shown in Figure 6.

**Figure 7:**
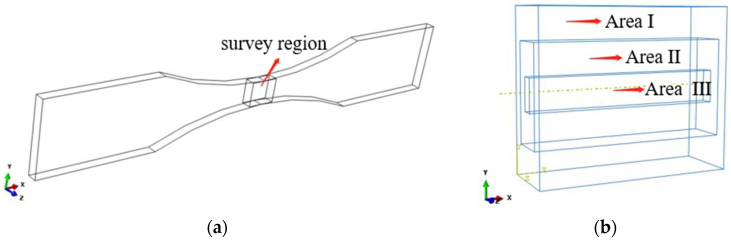
Regional processing of model. (**a**) Middle part of the study area, (**b**) subregion processing.

The stress in the middle area of the finite element model is large, and the strain is obviously seen from the stress–strain diagram of the specimen. The stress tends to increase gradually from the middle to the edge. Due to the influence of stress distribution, the damage inside and outside the material is also different. Therefore, the influence of different regions from inside to outside should be considered in the study of fatigue life. Therefore, a local analysis is carried out on the middle rectangular region with a size of 2 mm×5 mm×6 mm, as shown in Figure 7a. The intermediate region is separately divided into three specific regions from the inside out, namely Area 1, Area 2 and Area 3, as shown in Figure 7b.

### 3.3. Model Building

As established through a large number of experimental studies, the radius of the holes is in the range of 3–7 μm. A certain number of holes are randomly generated in region 1, region 2, and region 3, respectively.

**Figure 8:**
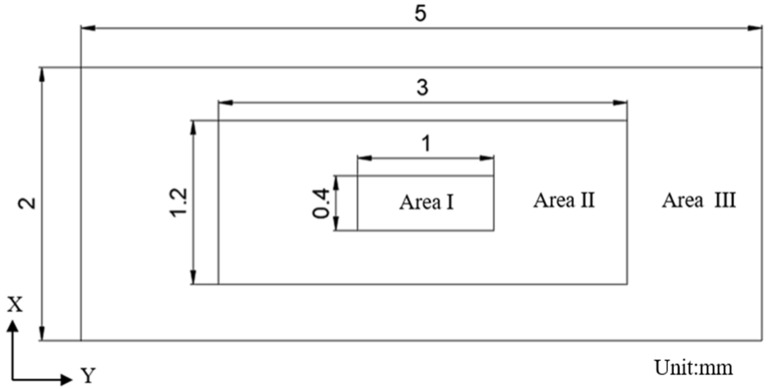
Model schematic diagram.

In the actual simulation process, it is found that the quality of mesh division will be very poor when the number of holes is 300. The calculation accuracy cannot be guaranteed. Therefore, five groups of simulation experiments are set up, and the number of holes is set as 50, 100, 150, 200 and 250, respectively. The random distribution of holes in each region is determined according to the coordinate points. The three-dimensional coordinates x, y, and z are set in different numbers with rectangular vertices as the origin. The holes are distributed according to the three areas $A_{1}$, $A_{2}$ and $A_{3}$ from inside to outside, as shown in Figure 8. The region division method is shown in Formulas (2)–(4).

(2) $$A_{1}=\left\{\begin{array}{c} 0.8<x<1.2\\ 2<y<3\\ 0<z<6 \end{array}\right.\;\mathrm{mm},$$

(3) $$A_{2}=\left\{\begin{array}{c} 0.4<x<1.6\\ 1<y<4\\ 0<z<6 \end{array}\right.-A_{1}\;\mathrm{mm},$$

(4) $$A_{3}=\left\{\begin{array}{l} 0<x<2\\ 0<y<5\\ 0<z<6 \end{array}\right.-A_{2}-A_{1}\;\mathrm{mm},$$

**Figure 9:**
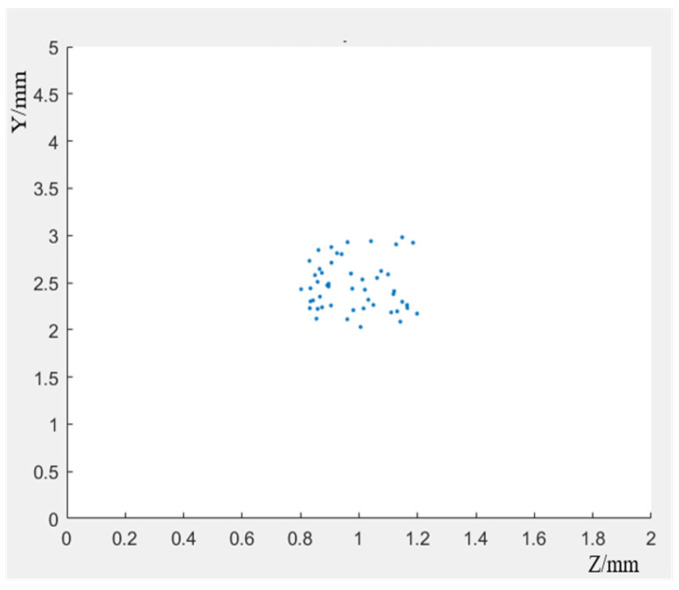
Hole distribution in region 1.

**Figure 10:**
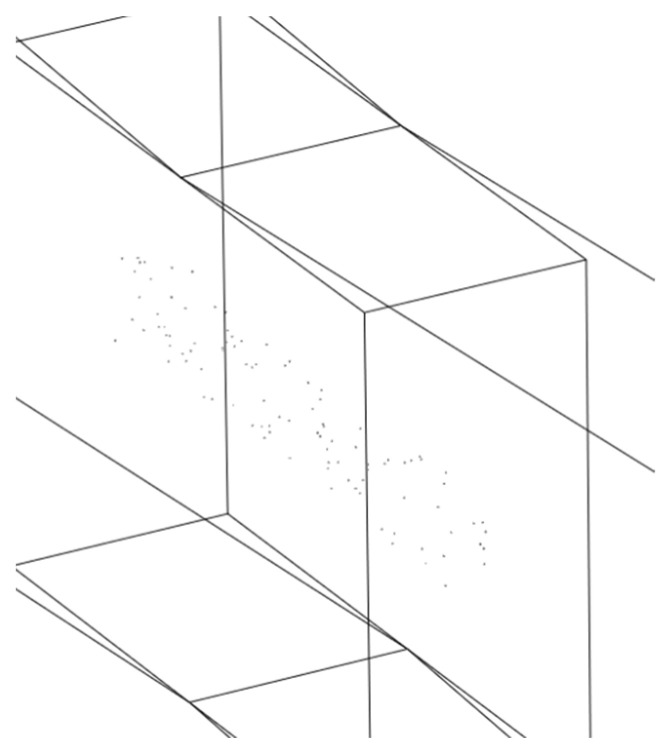
One hundred hole defect models in region II.

The generation of random points is realized through the linear congruence method of unified function in MATLAB. Each coordinate corresponds to a hole’s location information. The 50 holes are distributed in area 1, as shown in Figure 9. These holes are cut into the original model through the script of ABAQUS, which shows the creation of 100 hole models of the same size in region 2, as shown in Figure 10.

## 4. Result and Analysis

### 4.1. Fatigue Life under Random Holes

#### 4.1.1. Effect of Number of Holes on Fatigue Life

**Figure 11:**
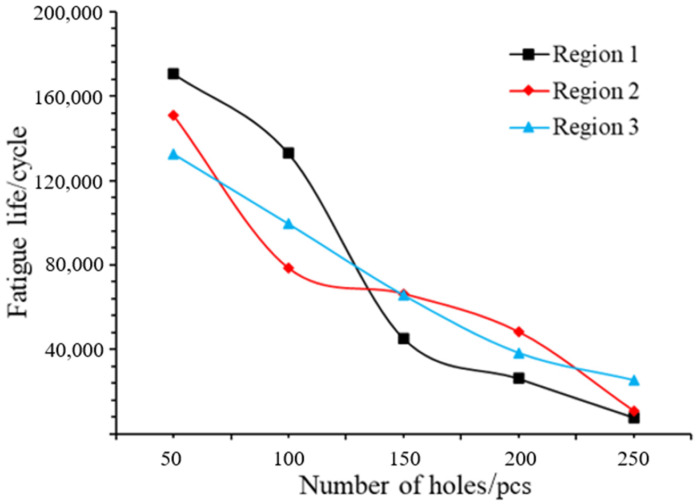
The curve of the overall fatigue life with different number of holes in each region.

The relationship between the number of random holes and the fatigue life is obtained through FE-SAFE analysis, as shown in Figure 11. It can be seen that the life of the specimen decreases with the increase in the number of holes. The fatigue life also decreases when the holes are distributed from region 1 to region 3, and the number of holes is about 50. This is because the positions of holes move from the inside to the surface, and the outer surface is prone to forming micro-cracks. The fatigue life is almost the same when the number of holes in the three regions is about 130. From the overall trend, the fatigue life fluctuation of region 1 is the largest with the increase in the number of holes, and the difference can reach 16,299 cycles. This is due to the fact that region 1 is located in the internal center and is prone to stress concentration, resulting in internal damage failure.

#### 4.1.2. Effect of Hole Size on Fatigue Life

**Figure 12:**
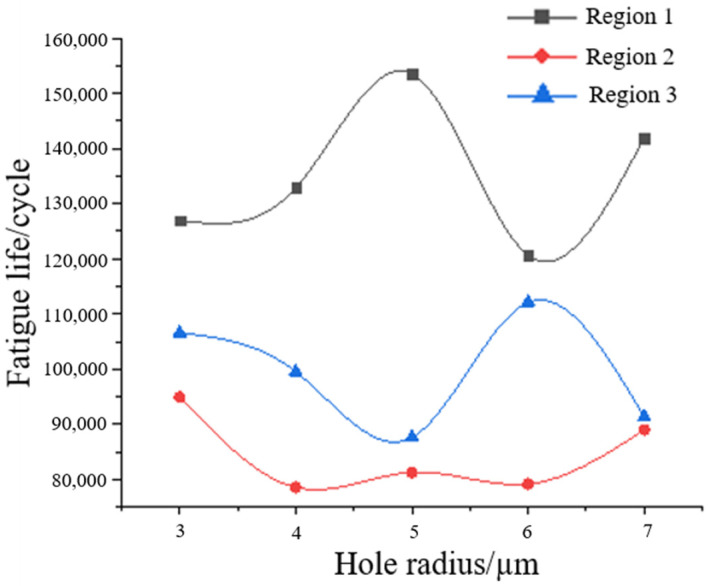
Fatigue life curve with the hole radius.

Under the premise of 100 holes in each region, the change trend of hole size and fatigue life is obtained through FE-SAFE, as shown in Figure 12. It can be seen from the figure that the fatigue life of the specimen does not change significantly with the increase in the radius of the hole. The holes produced in the fatigue damage are far smaller than the holes formed during the preparation of the material, which are at the micron level. This level of hole size often struggles to have a greater impact on the overall life of the material. It is generally believed that the pore size of metal materials is between 25 and 100 μm [10]. This is also verified by the fact that the fatigue life studied in this paper has no obvious change with the increase in hole radius.

### 4.2. Fatigue Life of the Micro-Crack Stage

The fatigue life of general materials is obtained according to the *S*–*N* curve, and it is expressed by the power function [30], as shown in Formula (5).

(5) $$\sigma^{m}N=C$$

(6) $$N=C\sigma^{-m}$$

(7) σ=An

(8) $$N=C{\left(An\right)}^{-m}=kn^{-m}$$

where σ is the stress amplitude or maximum stress; N is the number of cycles during fatigue failure; m and C are material constants; and A is the scale factor. According to Formula (8), the hole data in Table 3 are fitted by the least square method, and the functional relationship between fatigue life N and the number of holes $n_{i}$ can be obtained, as shown in Formulas (9)–(12).

(9) $$N_{1}=f(n_{1})=1209{n_{1}}^{-1.073}$$

(10) $$N_{2}=f(n_{2})=636.8{n_{2}}^{-0.9545}$$

(11) $$N_{3}=f(n_{3})=298.1{n_{3}}^{-0.7832}$$

(12) $$N_{i}=f(n_{i})=A{n_{1}}^{-B}$$

From the above formula, it can be inferred that there is a similar functional relationship between the fatigue life and the number of holes in the three regions. However, there is a certain relationship between region i and the coefficients A and B, and Formulas (13) and (14) are obtained after fitting, where i is 1, 2 and 3.

(13) A=−455.45i+1626.2

(14) B=−0.1449i+1.2267

Due to the difference in the number of holes in different regions, it is necessary to obtain the influence of holes in different regions on the overall life. It can be seen from the above relationship that the overall fatigue life N can be obtained by the product of regional fatigue life $N_{i}$ and life proportional coefficient S when the number of holes is the same. The determination of S is based on the premise that the number of holes in the preceding text is the same, where $n_{1}=n_{2}=n_{3}$. The value of $S_{i}$ can be determined as follows (15)–(17):

(15) $$S_{1}=\frac{N_{1}}{N}=0.05231{n_{1}}^{0.6877}$$

(16) $$S_{2}=\frac{N_{2}}{N}=0.099{n_{2}}^{0.5692}$$

(17) $$S_{3}=\frac{N_{3}}{N}=0.2122{n_{3}}^{0.3979}$$

In summary, the fatigue life of the specimen at the crack initiation stage can be calculated by adding the proportion of the life of different regions, so the final formula of random holes life of any region of the complete specimen is (18)

(18) $$N_{m}=\sum_{i=1}^{3}P_{i}f(n_{i})S_{i}$$

where P is the proportion of holes in the regions; S is the proportional coefficient of life; and n indicates the number of holes. The value of i is 1, 2, and 3.

### 4.3. Fatigue Life of Crack Growth Stage

After analyzing the microscopic failure life of the material, the residual life of the material with different hole distributions can be obtained. These lifetimes represent the initiation of macro-crack growth in materials. Therefore, it is necessary to analyze the macro-crack growth after crack forming to calculate the complete fracture life.

**Figure 13:**
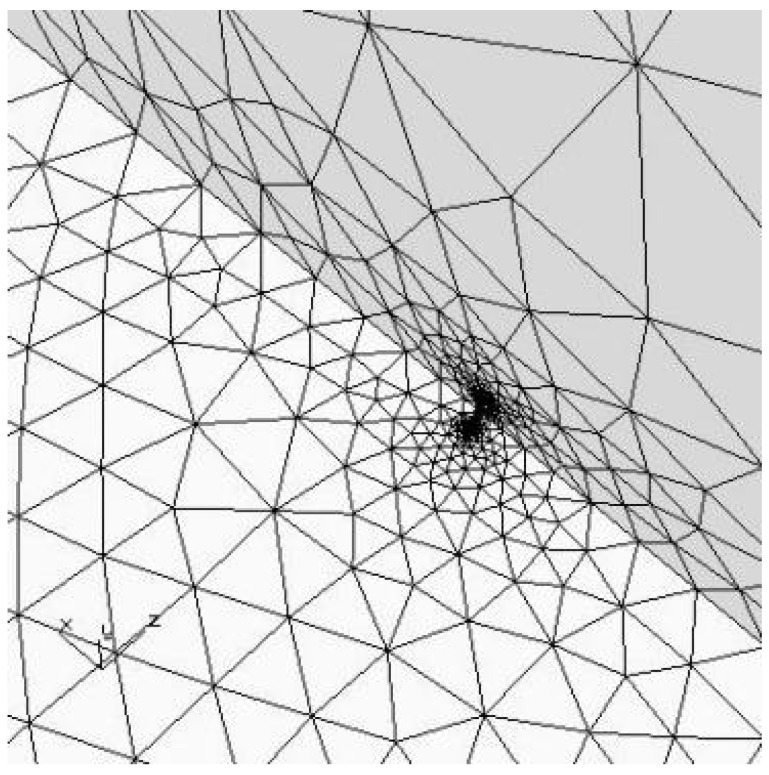
Layout of cracks at failure points.

In this paper, FRANC3D software is used to analyze the fatigue crack growth life. The material parameters of 6061-T6 aluminum alloy are built in the software. Considering that the crack initiation is located at the maximum stress of the sharp angle of the material, and the length of the crack macro growth stage is 0.01 mm < a < a_c_ (a_c_ is the critical crack size), the crack is set to an elliptical crack with a long half axis of 0.1 mm and a short half axis of 0.05 mm, as shown in Figure 13. The existence of the hole will affect the overall strength of the material, and the stress at the crack tip is much larger than the stress at the hole. Therefore, three models of hole numbers (0, 50, 100) were set up for the comparative study of crack growth.

**Figure 14:**
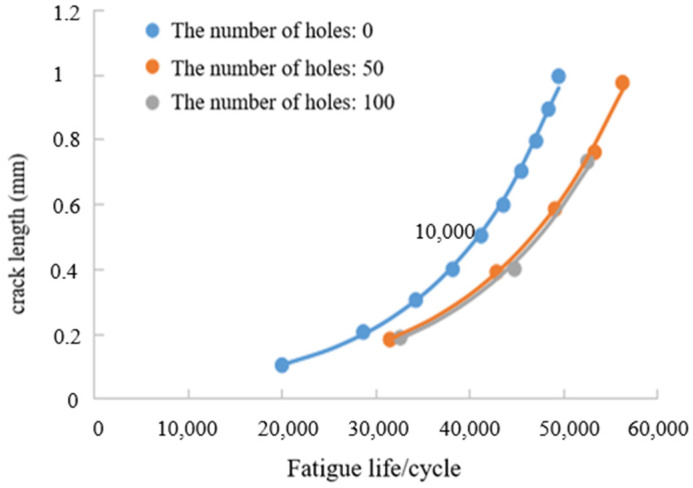
Fatigue crack growth length under different hole numbers.

Figure 14 shows the crack length with the number of holes. It can be seen that the crack growth life increases slightly with the increase in the number of holes. This is due to the fact that the stress intensity factor at the crack tip decreases when the number of holes at the crack tip increases. At this time, the crack growth rate will also decrease, which will prolong the fatigue life.

As the holes will have a tendency to merge into large holes with continuous cyclic loading, the number of holes will stop increasing when it exceeds a certain critical value. Therefore, in this paper, only three kinds of holes in Figure 13 are selected, and the curve is fitted by MATLAB to obtain the functional relationship between fatigue life n and crack length $L_{i}$, using Formulas (19)–(21). The width of the specimen is 5 mm, so the maximum crack length $L_{i}$ is 5 mm in the formula. Therefore, the fatigue crack growth life under three kinds of hole distribution can be obtained, as shown in Table 4.

(19) $$L_{0}=0.0223\;e^{0.00008N}$$

(20) $$L_{50}=0.0219\;e^{0.00007N}$$

(21) $$L_{100}=0.0257\;e^{0.00006N}$$

According to the data of the above three groups of data fitting, the life function of fatigue crack growth is obtained, as in Formula (22).

(22) $$N_{L}=2472.1\ln(n)+67685$$

where $N_{L}$ is the lifetime of crack growth and n indicates the number of holes. 

### 4.4. Lifetime Prediction and Verification

The fatigue failure of aluminum alloys undergoes two stages, namely crack initiation and crack growth. Therefore, the fatigue life of the whole stage is the sum of the two parts.

#### 4.4.1. Crack Initiation Stage

The accuracy of the CT scan results obtained by the three-dimensional visual software AVIZO is 3 μm. In the actual random hole analysis, 5958 holes with a radius greater than 3 μm were retained to screen the size and coordinate information of the hole defects with 40,000 cycles. The proportion of holes in region 1, region 2 and region 3 is 3.1%, 43.2% and 53.6% through the statistical analysis of the location information of all holes. The fatigue life of the crack initiation stage, calculated using Formula (18), is 30,999, as shown below. In addition to the previous 40,000 prefab crack cycles, the material displayed macro-cracks at about 70,999 cycles.

$$N_{m}=\sum_{i=1}^{3}P_{i}f(n_{i})S_{i}=30999$$

The characteristic parameters of holes can be seen through the fatigue test. There are only some small holes, and the changes are gentle when cyclic loading is performed 40,000 times, as shown in Table 5. After 80,000 cycles, the number of holes begins to decrease and microcracks appear inside the material. This is because the holes and cracks begin to fuse with each other, and the cracks begin to enter the growth phase, as shown in Table 6.

#### 4.4.2. Macro-Crack Growth Stage

In order to obtain the growth life of macro-cracks, it is necessary to consider the initial 40,000 loading cycles and fatigue cycles of crack initiation. Therefore, the growth life of macro-cracks was analyzed through cyclic loading 80,000 times. According to Table 6, the average number of holes is 8654 when loading 80,000 times. According to Formula (22), the growth life of macro-crack $N_{L}$ is 90,096, as follows:

$$N_{L}=2472.1\ln(n)+67685=90096$$

The whole fatigue life is estimated to be 161,095 from crack initiation to crack growth, as shown in Formula (23).

(23) $$40000+N_{m}+N_{L}=161095$$

In addition, the same three specimens were selected as the control group, and the whole life model was verified via experiments. The comparison between the experimental value and the theoretical prediction value of the whole life is shown in Table 7. The comparative analysis shows that the error is about 10%. Due to the uncertainty of the experiment, the error is within a reasonable range.

In addition, the life prediction models of Hao Chen and Chao Wang et al. [13,15] were selected as the control groups to further verify the reliability of the fatigue life prediction model proposed in this paper. Their theoretical models take the stress concentration coefficient and stress intensity factor as intermediate variables, respectively. A large number of hole parameters were trained via the SVM and PINN algorithms to obtain the corresponding relationship between the hole parameters and the intermediate variables. Finally, the functional relationship between the intermediate variable and the fatigue life was established under different test stages (20,000, 40,000, 60,000, 80,000 and 100,000 times). The verification errors of the fatigue life models of the two theoretical models at different stages were obtained, as shown in Table 8. The average error of the model in this paper is close to the other two papers. The three models are essentially based on the internal defects of materials to establish different damage evolution models in order to achieve the purpose of fatigue life prediction. Therefore, the model in this paper is proved to be reasonable and effective again.

## 5. Conclusions

In this paper, the defect characteristic information of 6061-T6 aluminum alloy was extracted and analyzed through experiments. A whole life prediction model was established based on the random hole method from mesoscopic initiation to macro-crack growth. Based on the above research, the following conclusions are obtained:

(1) The defect characteristic information of the specimen under cyclic load was obtained through CT scanning technology. It was found that the number of holes decreased with the increase in the number of cyclic loads, and the number of holes reached a peak at 80,000 cycles. However, other pore parameters (porosity, surface area, radius) were positively correlated with the number of cyclic loads.

(2) A random hole model is established according to the characteristics of damage hole distribution.

(3) In the crack initiation stage, the functional relationship between the number and proportion of holes and the fatigue life was obtained through the joint simulation analysis of ABAQUS and FE-SAFE.

(4) In the crack growth stage, the crack growth rate under different numbers of holes and loading cycles is obtained through FRANC3D. The crack propagation rate decreased with the increase in the number of holes. The functional relationship between the random holes and the number of cycles was established by means of numerical analysis.

(5) A lifetime model with random holes as damage variables was established based on the stages of microscopic crack initiation and macroscopic crack growth. The experimental comparison verifies the rationality of the model. It also provides a new ideas for the fatigue life prediction of aluminum alloys.

## Figures and Tables

**Table 1:** Composition of 6061-T6 aluminum alloy made in the United States.

Element	Cu	Mn	Mg	Zn	Cr	Ti	Si	Fe	Al
Inclusion component (%)	0.2~0.4	0.15	0.8~1.2	0.25	0.3	0.15	0.4~0.8	0.7	95.8

**Table 2:** Mechanical parameters of aluminum alloy 6061-T6.

Mechanical Parameter	Value	Unit
Tensile strength	310	MPa
Elastic modulus	69	GPa
Poisson’s ratio	0.33	/
Density	2.7	g/cm^3^
Yield strength	240	MPa

**Table 3:** The relationship between the number of holes and fatigue life in different areas.

Number of Holes		50	100	150	200	250
Fatigue life/cycle	Region 1	170,807	132,895	45,233	26,230	7816
	Region 2	151,038	78,526	66,389	48,253	10,704
	Region 3	132,649	99,476	65,623	38,221	25,318

**Table 4:** Fatigue crack growth life under different holes numbers.

Number of Holes	0	50	100
Fatigue crack growth life *N*_L_/cycle	67,651	77,581	87,845

**Table 5:** Maximum radius of holes at different stretching stages.

Cycle Index (10^4^)		4	8	12	16
Maximum hole radius/μm	Group I	6.52	9.777	16.779	39.425
	Group II	6.436	12.524	15.781	37.189
	Group III	6.628	11.406	12.654	26.919

**Table 6:** The number of holes at different stretching stages.

Cycle Index (10^4^)		4	8	12	16
Number of holes	Group I	5958	8104	3256	2563
	Group II	6541	7445	4952	3652
	Group III	7412	10,113	4562	2814

**Table 7:** Comparison of the total cycle life of the fractured specimens.

Specimen	1	2	3
Experimental fatigue life	180,236	177,426	168,652
Theoretical prediction of fatigue life	161,095	161,095	161,095
Error/%	10.6	9.2	4.5

**Table 8:** Comparison of fatigue life prediction models.

Testing Stage/Cycle		20,000	40,000	60,000	80,000	100,000	Average Error
The error between theory and experiment (%)	Hao Chen’s theory [13]	4	6.3	3.8	5.1	5.2	4.88
	Chao Wang’ theory [15]	10	6.6	2.3	2	2.5	4.68
	The theory of this paper	/					8.1

## Data Availability

The data that support the findings of this study are available within the article.
